# Effects of coenzyme Q10 supplementation on C-reactive protein and homocysteine as the inflammatory markers in hemodialysis patients; a randomized clinical trial

**DOI:** 10.15171/jnp.2016.07

**Published:** 2015-11-07

**Authors:** Narges-Sadat Zahed, Maryam Ghassami, Hajar Nikbakht

**Affiliations:** ^1^Departement of Nephrology, Loghman Hakim Hospital, Shahid-Beheshti University of Medical Sciences, Tehran, Iran; ^2^Department of Internal Medicine, Loghman Hakim Hospital, Shahid-Beheshti University of Medical Sciences , Tehran, Iran

**Keywords:** Homocysteine, C-reactive protein, Renal insufficiency, Coenzyme Q10

## Abstract

*Background:* The most leading cause of death in end-stage renal disease (ESRD) patients are cardiovascular disease and inflammatory markers are related to coronary events. CO-Q10 (coenzyme Q10) is a protective supplement from free radical oxidative damage. In addition, hyperhomocysteinemia is an independent coronary artery disease (CAD) risk factor.

*Objectives:* Due to increasing oxidative stress in dialysis patients, and the effect of CO-Q10 in decrease oxidative stress, in this work, we assessed the effect of CO-Q10 on C-reactive protein (CRP) level as an inflammatory marker and homocysteine in dialysis patients.

*Patients and Methods:* This was a single-blind, randomized cross over clinical trial. Patients with ESRD were randomly allotted to two groups. All patients received placebo and C0- Q10 100mg/d during the three months in each stage, with two week washout period. Plasma level of CRP and homocysteine from the start of the work and at the conclusion of each menses, are evaluated.

*Results:* Thirty-four patients randomized, but 26 patients complete study protocol. The treatment effect of CO-Q10 on CRP level is significant (*P* < 0.001) (95% CI = -20.1 to -10.5) and it was also significant for the increasing albumin level. (*P* = 0.044) (95% CI = 0. 0-0.6), But there was not any substantial effect on serum homocysteine level (*P* = 0.630).

*Conclusions:* CO-Q10 could significantly decrease CRP level as an inflammatory marker and can protect cardiovascular events.

**Implication for health policy/practice/research/medical education:**

The most leading cause of death in end-stage renal disease (ESRD) patients are cardiovascular disease and inflammatory markers are related to coronary events. Coenzyme Q10 (CO-Q10) is a protective supplement from free radical oxidative damage. In addition, hyperhomocysteinemia is an independent coronary artery disease (CAD) risk factor.Our study shows coenzyme Q10 reduced the C-reactive protein (CRP) as an inflammatory marker amongechronic renal failure patients.Inflammatory markers are supposed to result in many different complications. The authors hope, the results of the present study help to improve the complication of chronic renal failure patients patients.

## 1. Background

In patients with chronic kidney disease (CKD), one of the important comorbidities which may lead to mortality and morbidity is cardiovascular events (1,2). Role of oxidative stress in coronary artery disease (CAD) is well established (2). On the other hand, in CKD patients other than traditional risk factors such as hyperlipidemia, other risk factors like oxidative stress and inflammation are attributed to elevated cardiovascular event rate (3,4). High levels of inflammatory markers like high-sensitivity C-reactive protein (hs-CRP) is able to predict risk of myocardial infarction (5). Elevated oxidant markers like hs-CRP are also detected in most of CKD patients as a sign of inflammation (6). Another detectable marker in inflammation and pathogenesis of CVD is homocysteine (7). Coenzyme Q10, is one of intracellular antioxidant components, protect cell membrane and mitochondrial protein from oxidative stress (8-10). Anti-oxidants like coenzyme Q10 is thought to reduce serum oxidant levels. While clinical trials studies, examined the effect of coenzyme Q10 in CVD patient (11,12), to our knowledge, there are few studies assessing the effect of coenzyme Q10 in CKD patients (13-15). In addition few recent studies look into the effect of coenzyme Q10 on inflammation and oxidative markers in CVD patients (16-18). Additionally, the possible relationship between coenzyme Q10 and oxidative stress markers in CKD patients was not been studies completely (19).

## 2. Objectives

In the current study, we aimed to evaluate the effect coenzyme Q10 on some inflammatory markers including hs-CRP and homocysteine in patients with CKD.

## 3. Patients and Methods

This is a single-blind, randomized cross over clinical trial. We studied 34 end-stage renal disease (ESRD) patients undergoing dialysis in Ashrafiesfehsni and Loghman-Hakim hospitals in Tehran from November 2013 to September 2014. From 373 patients referring to studied centers, 34 patients who fulfilled the inclusion criteria were enrolled. The inclusion criteria were acceptation to participate in the study, ESRD patients who were under dialysis for at least 8 months, blood pressure below 180/100 mm Hg, no smoking, patients whom, on folic acid therapy for at least 6 months before the study, absence of any active or chronic disease, hs-CRP more than 10 mg/L and finally highs level of homocysteine according to laboratory reference range prior to the study. Exclusion criteria were age under 18 years, cirrhosis, active neoplasm, post-menopausal women who are under hormone replacement therapy and hospitalization due to acute infection, acidosis or toxic shock. Before the clinical trial, age, level of blood pressure, Kt/V, level of hs-CRP and homocysteine were checked in all patients whom enrolled to the study. Thirty-four patients with hs-CRP >10 mg/L and high homocysteine level according to laboratory cut off were enrolled and divided into A and B groups by table of random numbers. Patients were not cognizant of their group allocation. Each patient should take one capsule a day as placebo or drug according to the randomization. In this study, group A was Q10-placebo and group B, regarded as placebo-Q10. During first three months of study patients in group A were administered CoQ10 (Health Burs Co) 100 mg once daily and patients in group B were administered placebo for 3 months. After 2 weeks of washout period patients in group A were administered placebo and in group B were administered CoQ10 for another 3 months with the same protocol. Quantitative measurement was applied to determine the hs-CRP levels and plasma homocysteine were measured using high-performance liquid chromatography (HPLC). Patients were closely observed by a physician during the study.

### 3.1. Ethical issues

1) All procedures performed in studies involving human participants were in accordance with the ethical standards of the institutional and/or national research committee and with the 1964 Helsinki declaration and its later amendments or comparable ethical standards; 2) informed consent was obtained, they were free to leave the study at any time and; 3) research was approved by the ethical committee of Shahid-Beheshti University of Medical Sciences (Research proposal number: 297).

### 3.2. Statistical analysis

Data are extracted as a mean ±SD or as median values. *T* test, Fisher exact test and chi-square test was used to compare normally distributed continuous data and categorical information. Mann–Whitney U test was applied to data that were not normally distributed. By Pocok method, carry over effect was examined. Linear mixed model (LMM) was applied to evaluate carry-over, period effect and treatment outcome. Period effect is the result of other treatments or the typical disease development during the study period. The treatment effect is the effect of intervention used in the study. The level of significance was *P *< 0.05 for all compared variables. Data were analyzed using SPSS version 22 (SPSS Inc., Chicago, USA).

## 4. Results

**Figure 1:**
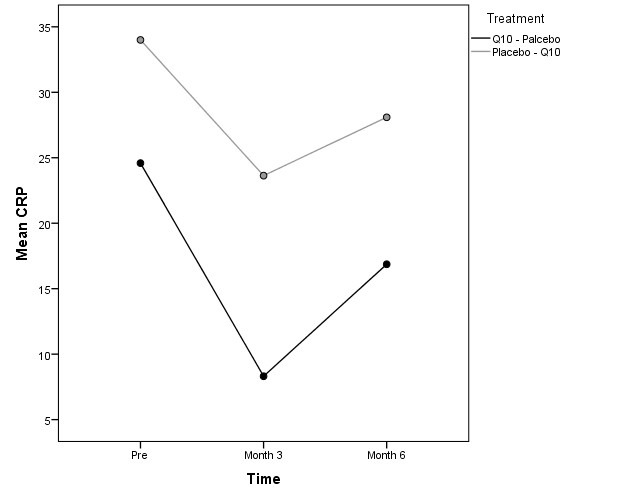
Effect of Q10 on mean CRP levels. Q10 supply have statistically significant effect on the reduction of hs-CRP (*P* < 0.001).

**Figure 2:**
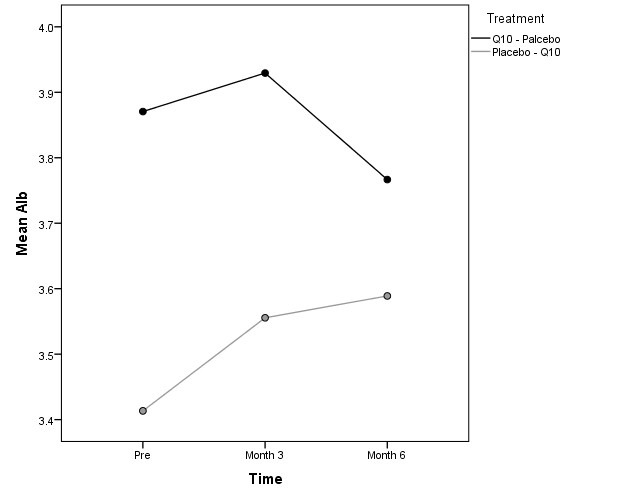
Effect of Q10 on mean serum albumin level. Serum albumin was significantly increased after treatment (*P* = 0.04).

Of 34 patients, 8 patients were not continued the study protocol. Two patients in group A died, one as the result of acute infection and the other patients by cardiovascular events. Six patients in group B (three due to hospitalization and three who died) were excluded from the study. Thus the study continued with 21 men and 13 adult females. There was not statistically difference between two groups of A and B according to gender, age, dialysis duration and Kt/V (Table 1). The etiologies of ESRD are described in Table 2. The average changes of parameters before and after intervention is shown in Table 3. There was not significant difference of systolic blood pressure (*P* = 0.740) and hemoglobin (*P* = 0.460) between two groups. In our study, after adjustments of statin impact, we found statistically significant carry-over effect for hs-CRP (P <0.001; Figure 1). The results of the carry-over effect for homocysteine did not show a significant reduction in two periods of treatments (*P* = 0.422). However period-effect was statically significant (*P* = 0.009). Moreover, treatment effect for homocysteine was not significant (*P* = 0.715). After adjustments of the effect of baseline characteristics and statin effect LMM the results for homocysteine was the same and the period effect on the serum homocysteine level was significant (*P* = 0.001). The treatment effect (*P* = 0.630) and carry-over effect was not of significant among the study groups (*P* = 0.400). In this study, serum albumin was increased significantly according to treatment effect (*P* = 0.04; CI 95% = 0. 0-0.6; Figure 2). Additionally, the carry-over-effect and period effect were not significant (Table 4).

**Table 1:** Patients’ baseline characteristics

**Baseline characteristics**		**Total**	**Group A**	**Group B**	**P value**
Age(years)	Mean±SD (range)	66.3±11.3 (43-90)	66.4±11.5 (43-90)	66.2±11.4 (46-89)	0.964
Gender	Male	21(61.8%)	11(64.7%)	10(58.8%)	0.724
	Female	13(38.2%)	6(35.3%)	7(41.2%)	
Dialysis (weeks)	Mean±SD (range)	47.2±40.2 (8-180)	43.6±39.7 (14-180)	52.7±42.2 (8-156)	0.611
Kt/V	Mean±SD (range)	1.2±0.4 (0.54-2.36)	1.22±0.48 (0.54-2.36)	1.19±0.25 (0.83-1.7)	0.850
Statin supply	Yes	10(29.4%)	3(17.6%)	7(41.2%)	0.132
	No	24(70.6%)	14(82.4%)	10(58.8%)	

**Table 2:** The etiology of ESRD among study group

	**Group A (Q10-placebo) n (%)**	**Group B (placebo-Q10) n (%)**	**P value**
Diabetes mellitus	9 (52.9%)	5 (29.4%)	0.065
Hypertension	6 (35.3%)	3 (17.6%)	0.065
Idiopathic	2 (11.8%)	8 (47.1%)	0.065
Glomerulonephritis	0 (0%)	1 (5.9%)	0.065

**Table 3:** Average of parameters before and after intervention

**Parameters**		**Week 0**	**First 3 months**	**Second 3 months**
Systolic blood pressure (mm Hg)	A	129±23	127±22	125±23
	B	133±13	132±14	132±16
Hemoglobin (g/dl)	A	10.76±1.6	10.49±1.76	11.11±2.11
	B	10.31±1.11	10.3±0.87	10.3±1.26
hs-CRP (mg/dl)	A	24.6±11.7	8.3±7.2	16.9±15
	B	34±15.6	23.6±3.2	28.1±21.1
Homocysteine (μmol/l)	A	25.1±5.4	32±11.8	15.7±5.2
	B	25.4±12.2	33.6±8.37	21.2±5.8
AST (IU/l)	A	15±7	20±7	18±6
	B	22±6	24±7	28±9
ALT (IU/l)	A	19±10	18±8	16±6
	B	19±7	18±6	20±13
Albumin (g/dl)	A	3.87±0.58	3.93±0.54	3.77±0.34
	B	3.41±0.27	3.56±0.37	3.59±0.38

**Table 4:** Parameter analysis after Co-Q10 supplementation

	**Carry-over-effect**	**Period-effect**	**Treatment-effect**
Systolic blood pressure (mm Hg)	0.286	0.736	0.740
Hemoglobin (g/dL)	0.793	0.453	0.460
CRP (g/dL)	0.013	0.063	<0.001
Homocysteine (μmol/l)	0.659	0.001	0.630
AST (IU/l)	0.882	0.245	0.064
ALT (IU/l)	0.215	0.860	0.157
Albumin (g/dl)	0.378	0.184	0.04

## 5. Discussion

Inflammation is highly associated with complications and cardiovascular adverse events in CKD. In the current study, we found coenzyme Q10 (100 mg/day) reduced serum hs-CRP levels as an inflammation marker. However homocysteine levels were not influenced by coenzyme Q10 supplementation. It should be noted that the period effect for homocysteine was significant. In our study blood pressure was not reduced significantly which was in accordance with the results of study by Mori et al (20). However Shargorodsky et al showed coenzyme Q10 could reduce blood pressure and increase elasticity in small and large vessels in non-CKD patients, with multiple cardiovascular risks (21). In 2013 another cross-over study suggested coenzyme Q10 did not have a favorable impact on diastolic heart function in hemodialysis patients (22). One clinical trial on CVD patients reported, coenzyme Q10 reduced IL-6 plasma levels and did not have coloration with hs-CRP and homocysteine (17). Lee et al in the other study suggested that coenzyme Q10 supplementation at the dose of 150 mg/day result in rapid and sustain a decrease in oxidative stress among CVD patients in comparison with healthy people (18). This effect on not healthy population may attribute to the highest levels of inflammatory markers in patient than in healthy people. In agreeing with Lee et al study, Gokbel et al shows coenzyme Q10 (100 mg/day) in healthy population did not affect the inflammatory markers (19). The above mentioned dose seems not to be sufficient to reduce inflammatory markers which were not elevated among healthy people. Although in dialysis patients, the serum oxidative markers is high (23), some other investigations could not obtain the favorable effect of coenzyme Q10 therapy on hs-CRP, homocysteine and IL6 (21,22). Among patients with breast cancer who were under treatment of coenzyme Q10 (100 mg/day) and vitamin B group (riboflavin 10 mg and niacin 50 mg), inflammatory markers included TNF-α, IL-8, IL-6, IL-1β were significantly decreased (24). Since all participants in our study used folic acid as a treatment of hyper homocysteinemia (25), period effect on homocysteine can be justified as folic acid effect. Furthermore albumin plasma levels as an effective factor for better surveillance of the patients, raised in our study.

## 6. Conclusions

Co-Q10 supplement could reduce hs-CRP levels significantly, which is a known inflammatory factor. Co-Q10 could raise serum albumin level as a marker of better surveillance factor. According to the favorable effect of CoQ10 on Co-Q10 level, this antioxidant may play the role in reducing CVD events in CKD patients.

## 7. Limitations of the study

The limitations of our study was small sample size and short duration of investigation. Due to confidence outcomes, more studies with larger sample size and in a longer period of time are recommended.

## Acknowledgments

We would like to acknowledge the clinical research development center of Loghman-Hakim hospital for its help.

## Authors’ contribution

NSZ designed the study and observed accuracy and validity of study protocol. MG collected the data and followed the studies objects. HN wrote the article and edited the manuscript.

## Conflicts of interest

The authors declared no competing interests.

## Funding/Support

None.
